# Supplementary material for: Empirical methods for controlling false positives and estimating confidence in ChIP-Seq peaks
Source: BMC Bioinformatics. 2008 Dec 5;9:523. doi: 10.1186/1471-2105-9-523 (PMC2628906; doi:10.1186/1471-2105-9-523)
Supplement: Additional File 1 — A variety of html documents from the USeq web site detailing the available applications, their best usage, output file type descriptions, command line menus, etc. [file 1471-2105-9-523-S1.zip › USeqUserGuides/JavaDoc.zip!/JavaDoc/gata/main/class-use/AnnoSpecParams.html]

Uses of Class gata.main.AnnoSpecParams


|  |  |  |
| --- | --- | --- |
| **Overview** \| **Package** \| **Class** \| **Use** \| **Tree** \| **Deprecated** \| **Index** \| **Help** | |  |
| PREV   NEXT | **FRAMES**    **NO FRAMES**     **All Classes** |  |


---


## **Uses of Class gata.main.AnnoSpecParams**

| Packages that use AnnoSpecParams | |
| --- | --- |
| **gata.geneGlyphs** | For representing annotation, scale bars, and generic tracks. |
| **gata.main** | Main app, utility, and params for GATAPlotter. |
| **gata.menu** | GATAPlotter menus, panels, and frames. |
| **gata.plotter** | GATAPlotter primary panel, sliders, tools, and conservation panels. |

Uses of AnnoSpecParams in gata.geneGlyphs

Constructors in gata.geneGlyphs with parameters of type AnnoSpecParams

`Annotation(AnnoSpecParams annoParams,
GATAParams gataParams)`

`GeneRepGlyph(GeneRep geneRep,
GATAParams gataParams,
AnnoSpecParams annoParams)`

`GlyphPanel(AnnoSpecParams annoParams,
GATAParams gataParams)`

`ScaleBar(AnnoSpecParams annoParams,
GATAParams gataParams)`

`TransGrpGlyph(TransGroup transGrp,
GATAParams gataParams,
AnnoSpecParams annoParams)`

Uses of AnnoSpecParams in gata.main

| Methods in gata.main that return AnnoSpecParams | |
| --- | --- |
| `AnnoSpecParams` | `GATAParams.getCompAnnoSpecParams()` |
| `AnnoSpecParams` | `GATAParams.getRefAnnoSpecParams()` |

| Methods in gata.main with parameters of type AnnoSpecParams | |
| --- | --- |
| `void` | `GATAPlotter.makeAnnotation(AnnoSpecParams annoParams)` |
| `void` | `PlotterPreferences.setAnnoScaleDefaults(AnnoSpecParams asp)` |
| `void` | `GATAParams.setCompAnnoSpecParams(AnnoSpecParams params)` |
| `void` | `GATAParams.setRefAnnoSpecParams(AnnoSpecParams params)` |

Uses of AnnoSpecParams in gata.menu

| Methods in gata.menu with parameters of type AnnoSpecParams | |
| --- | --- |
| `void` | `GATAFrame.completeAnnotationMenu(boolean gffRefPres, boolean gffCompPres, AnnoSpecParams refParams, AnnoSpecParams compParams)`             must be called after the initial creation when the user has possibly loaded generic annotation |
| `void` | `GATAFrame.reallyRedrawAnnotationPanel(AnnoSpecParams asp)` |

Uses of AnnoSpecParams in gata.plotter

| Methods in gata.plotter with parameters of type AnnoSpecParams | |
| --- | --- |
| `void` | `AlignPanel.redrawGlyphPanel(AnnoSpecParams asp)` |

---


|  |  |  |
| --- | --- | --- |
| **Overview** \| **Package** \| **Class** \| **Use** \| **Tree** \| **Deprecated** \| **Index** \| **Help** | |  |
| PREV   NEXT | **FRAMES**    **NO FRAMES**     **All Classes** |  |


---
